# Tiny patients, huge impact: a call to action

**DOI:** 10.3389/fpubh.2024.1423736

**Published:** 2024-06-17

**Authors:** Jordee Wells, Anita Shah, Holly Gillis, Sarah Gustafson, Carmin Powell, Amornluck Krasaelap, Samantha Hanna, Jennifer A. Hoefert, Amee Bigelow, Jennifer Sherwin, Emilee C. Lewis, Katherine E. Bline

**Affiliations:** ^1^Department of Pediatrics, Division of Emergency Medicine, Nationwide Children’s Hospital, The Ohio State University College of Medicine, Columbus, OH, United States; ^2^Division of Hospital Medicine, Cincinnati Children’s Hospital Medical Center, Cincinnati, OH, United States; ^3^Department of Anesthesiology, Division of Pediatric Anesthestiology, University of Minnesota, Minneapolis, MN, United States; ^4^Division of Pediatric Hospital Medicine, Harbor-UCLA Medical Center, David Geffen School of Medicine at UCLA, Los Angeles, CA, United States; ^5^Division of Pediatric Hospital Medicine, Stanford University School of Medicine, Stanford, CA, United States; ^6^Department of Gastroenterology and Hepatology, SeattleChildren’s Hospital, Seattle, WA, United States; ^7^Saint Louis University School of Medicine, SSM Health Cardinal Glennon Children's Hospital, St. Louis, MO, United States; ^8^The Heart Center, Nationwide Children’s Hospital, Columbus, OH, United States; ^9^Division of Cardiovascular and Thoracic Surgery,Duke University Medical Center, Durham, NC, United States; ^10^Division of Hospital Pediatrics, Department of Pediatrics,University of North Carolina School of Medicine, Chapel Hill, NC, United States; ^11^Division of Critical Care Medicine, Department of Pediatrics, Nationwide Children’s Hospital, The Ohio State University College of Medicine, Columbus, OH, United States

**Keywords:** children, public health, Medicaid, Medicare, pediatric health care

## Abstract

The continuation of high-quality care is under threat for the over 70 million children in the United States. Inequities between Medicaid and Medicare payments and the current procedural-based reimbursement model have resulted in the undervaluing of pediatric medical care and lack of prioritization of children’s health by institutions. The number of pediatricians, including pediatric subspecialists, and pediatric healthcare centers are declining due to mounting financial obstacles and this crucial healthcare supply is no longer able to keep up with demand. The reasons contributing to these inequities are clear and rational: Medicaid has significantly lower rates of reimbursement compared to Medicare, yet Medicaid covers almost half of children in the United States and creates the natural incentive for medical institutions to prioritize the care of adults. Additionally, certain aspects of children’s healthcare are unique from adults and are not adequately covered in the current payment model. The result of decades of devaluing children’s healthcare has led to a substantial decrease in the availability of services, medications, and equipment needed to provide healthcare to children across the nation. Fortunately, the solution is just as clear as the problem: we must value the healthcare of children as much as that of adults by increasing Medicaid funding to be on par with Medicare and appreciate the complexities of care beyond procedures. If these changes are not made, the high-quality care for children in the US will continue to decline and increase strain on the overall healthcare system as these children age into adulthood.

## Introduction

The 2024 Health of US Primary Care Report shows that 1 in 10 children in the United States (U.S.) do not have a primary care doctor and that number is steadily increasing (1). Too often parents struggle to find a pediatrician and medical home for their children because medical practices reach the maximum capacity of children they will serve that are covered by Medicaid. Private primary care pediatrician cite paperwork, low payment and capitation as primary drivers of low Medicaid participation (2). Even commercially insured children have also seen a roughly 15% drop in primary care visits in the last decade (3).These limitations prevent vulnerable children from accessing affordable, high-quality health care. Our current healthcare system operates under the incorrect assumption that children require an inconsequential investment of healthcare dollars compared to adults and overlook the health challenges that are distinct to children. Children have unique physiology and require a unique approach that differs from adults. In the following, we advocate for the development of a comprehensive national strategy to create policies that recognize the intrinsic value of children and prioritize their health and well-being.

Major representative organizations, such as the American Academy of Pediatrics and Children’s Hospital Association, promote changes to reform pediatric payment structures and to re-evaluate the perceived value of pediatric healthcare. However, there remains a significant lack of representation for pediatric care at the tables where cost and payment decisions are made (4, 5). Without significant changes to prioritize the health and well-being of children, quality care for children will continue to decline in the U.S. and lead to worsening disparities as these children age into adulthood. The significantly lower reimbursement from Medicaid and Children’s Health Insurance Program (CHIP), which cover almost half of children in the U.S., is a major contributor to decreasing access to medical personnel and equipment for children (6). As payment for pediatric care remains far below the standards for the same care provided for adults, access to quality pediatric healthcare continues to decline, particularly in rural areas (7, 8).

For all these reasons, we believe that child health financing policy should be a primary focus of the health care movement. A collaborative approach centering value-based care with all stakeholders—health systems, payors, government--is necessary to improve the care delivered to children and to optimize overall child health and well-being. In this article, we will (1) provide an overview of Medicaid and how it supports children, (2) describe the costs that are unique to the care of children, (3) illustrate the challenges children face related to access to care, (4) discuss the growing needs for the care of children with medical complexity, (5) identify the problems with procedure based Relative Value Units (RVU) and (6) argue for a call to action for policy change and investment toward care delivery transformation. Collectively, the health care system must commit to enhance representation and ensure that children are valued at least as much as that adults, ultimately fostering societal welfare.

## Medicaid covers almost half of children in the U.S. but is significantly under-funded

Medicare and Medicaid were jointly created in 1965 under the Social Security Amendments to provide health care insurance for two of our nation’s most vulnerable populations (9). Medicare would provide financial support for older adult adults, while Medicaid provided financial support to those with no or limited income. Medicare is federally administered to improve access and basic health care benefits for the older adult to live out their years in dignity, irrespective of which state they reside. Unlike Medicare, Medicaid is a jointly funded partnership between federal and state governments (10). Although Medicaid has baseline federal requirements, it is directly managed by individual states and allows for a wide range of eligibility requirements and coverage benefits among states (11). For most states, Medicaid is the largest expenditure in state’s budgets and financed by the states general revenue (12). The Medicaid and CHIP Payment and Access Commission highlights how the law designates that at least 40 percent of Medicaid must be financed by the state and up to 60 percent can be from local governments; therefore, the variation of Medicaid’s share among state’s budgets can largely depend on how the allocated dollars are measured (13). These variations in allocated funds have kept state Medicaid expenditures rather stable compared to an increase in federal funding over the last decade (12).

The separate evolutions of Medicare and Medicaid programs led to significant pay disparities between adult care providers and pediatric care providers (14). Due to the wide variation in state funding for Medicaid, Medicaid reimbursement can be up to 70% less than federally-funded Medicare reimbursement for the same diagnoses or procedures (15–17). Together Medicaid and CHIP provide health coverage for over 40 million children in the U.S (18). The stark contrast in Medicare and Medicaid payment models highlights the disadvantages to healthcare facilities and staff that care for Medicaid-covered patients, particularly children (19, 20). It is imperative for policies to prioritize children and ensure they have the most basic form of access to health care coverage.

## Children have healthcare requirements unique from adults

The framework used in current payment models is adult-centric, not family-centric, and undervalues the medical care of children. The underfunding of pediatric care creates an inequitable burden on families and leads to downstream effects on the family’s ability to afford other essential items (e.g., food, childcare) for their children. Over 33 million children in the United States belong to families with incomes ≤250% below the federal poverty level, and these families are most likely to report a financial burden due to medical care (21).The most common reasons for guardians to forgo care for their children are medical costs (54%) and difficulty obtaining an appointment (46%). These barriers are primarily due to the inadequate investment in children’s healthcare (22). The intersection of childhood poverty and inadequate funding of children’s healthcare is an urgent public health problem.

Funding and reimbursement should prioritize both preventative and reactive healthcare for children. Much of the preventative care that is unique to pediatrics prevents harm and disability, as well as prolongs and improves quality of life. One successful example of preventative care is the newborn screening program, which includes screening for conditions at birth, such as cystic fibrosis or sickle cell anemia. The newborn screening program allows for earlier diagnosis and life-saving treatment for children (23). Investment in vaccine development and implementation has also transformed healthcare and eradicated several previously fatal diseases in the United States (24). In contrast to the life-saving success stories, the lack of investment in other pediatric preventative care creates lasting negative impacts on our children and future generations. As pediatricians, we are now seeing alarmingly high rates in the early onset of diseases in children that historically started in adulthood; for example, obesity and its associated conditions such as type II diabetes (25, 26). Another consequence of the lack of investment in children is the significant rise of mental health related disorders (27). In the U.S., the number of pediatric mental health hospitalizations increased 25% between 2009 and 2019 (28). The worsening of pediatric mental health before and during the COVID-19 pandemic led to a call from the U.S. Surgeon General advisory to invest in youth mental health due to a significant rise in mental health emergencies in children (29). Investing early in pediatric preventative care can improve health outcomes as children age into adulthood and decrease future healthcare costs.

## Children have decreased access to healthcare

Access to healthcare for children in the U.S. is a patchwork system of payors including employer-sponsored coverage through parents, federal and state Medicaid and the Children’s Health Insurance Program (CHIP), Affordable Care Act (ACA) Marketplace plans, and other nongroup plans (30, 31). The U.S. healthcare systems prioritize the care of adults over the care of children due to profitability, and the lack of investment in children results in decreased access to pediatric care (32). From 2008 to 2018, pediatric inpatient units decreased by 34 units per year (19.1%). Rural areas were disproportionately affected, experiencing a 24.2% decrease in pediatric inpatient units and 26.1% decrease in pediatric inpatient beds (32). The majority of hospital closures occurred in states where Medicaid expansion did not occur (33). Although other factors, such as geographic isolation, regulatory barriers, and low patient volumes, also play a role in closure of rural hospitals, financial pressures remains the major driving force (33, 34). Consequently, interhospital transfer rates are increasing even for common conditions such as asthma exacerbations, abdominal pain, and gastroenteritis (35). The ED transfer rate for pediatric patients has increased over 25%, with the sharpest rise observed in low-volume and rural hospitals (36). The increasing transfer rate to pediatric facilities contributes to emergency department overcrowding at urban hospitals, which is associated with decreased patient safety, timeliness, and effectiveness of emergency care in children (37). There is also greater reliance on referral centers for children. Referral centers provide excellent pediatric care, but less than 250 children’s hospitals exist across the U.S. As the smaller facilities transfer more pediatric patients, this will lead to decreased clinical experience with pediatric care and further exacerbate children’s declining access to healthcare from experienced healthcare providers trained in pediatrics or pediatric management (36).

The shift away from treating pediatric patients in rural settings leads to the regionalization of care for children. Regionalization of care can contribute to decreased access to pediatric care in smaller and rural hospitals, resulting in unnecessary transfers for low-acuity conditions and increased cost to the health care system (36). Regionalization of care may also result in delayed diagnosis, increased travel time, lack of coordinated care, and difficulty with follow-up care for children (38, 39). An estimated 39 million children reside ≥80 miles from a pediatric subspecialist (40). Increased distance to healthcare is associated with decreased health care utilization, and children in rural areas are also more likely to receive care from an adult-trained subspecialist who is less experienced with disease presentation in children and evidence-based pediatric care (40). Additionally, children in rural areas have higher health care costs and increased readmission rates compared to non-rural children, further contributing to increased health care costs in this vulnerable population (39).

Multiple drivers contribute to the reduced access to pediatric care. The costs associated with maintaining pediatric-sized equipment and staffing a pediatric unit are prohibitive for many small hospitals to maintain, and specialized pediatric providers often migrate to larger academic centers (32). Additionally, Medicaid reimbursement rates are typically substantially lower than actual hospital costs, and a growing percentage of pediatric inpatient stays are covered by Medicaid rather than private insurance (6). In 2014, hospital financial losses from Medicaid underpayment totaled $14.1 billion. During the pandemic, many pediatric beds necessarily transitioned to adult care beds as adults were more severely affected by COVID-19. However, many institutions are reluctant to transition these beds back to pediatric care due to the better reimbursement of Medicare coverage for adult patients compared to decreased Medicaid reimbursement for the care of children.

## Children have increasing complexity of care

Pediatric providers and medical systems are increasingly more successful in managing conditions that were historically characterized by high mortality rates, e.g., extreme prematurity, liver failure, complex congenital heart disease, and childhood cancers (41–44). This improvement in the survival rate has led to increased medical complexity and morbidity for pediatric patients and created more demand for life-sustaining therapies. Advances in medical technology and home-based equipment, such as mechanical ventilation and nutritional support, contribute significantly to extending the survival of children. Over the last decade, hospitalizations for children with medical complexity (CMC) have surged by 33% (45). One major contributor to the increase in the number of CMC is the increased survival rate of extremely preterm neonates (gestational age 22–28 weeks), which is now as high as 78% (43). While an outstanding achievement, this increased survival rate is accompanied by substantial morbidities, including intracranial hemorrhage (14.3%) and severe bronchopulmonary dysplasia (8%) requiring specialized health needs at birth and additional needs as these children age. This patient population represents a major user of hospital resources, with one study finding that 49% were re-hospitalized by the 2-year follow-up and 21% exhibited severe neurodevelopmental impairment (43). The improvement in the life expectancy of CMC necessitates a greater allocation of time, resources, and specialized expertise to achieve optimal health and quality of life for this unique patient population.

The rise in medical complexity requires specialized providers, strains inpatient resources due to prolonged hospitalizations, and requires more frequent visits in outpatient settings. Pediatric healthcare institutions are responding by creating dedicated teams aimed at understanding, treating, and delivering resources for these children with complex needs to achieve better healthcare outcomes. Important markers of improved healthcare outcomes in this patient population include decreased hospitalizations and improved continuity of care (46). To achieve this, pediatric hospitals need to provide easy access to specialized providers for guidance and assistance to both families and healthcare professionals in remote areas. However, the high number of children covered by Medicaid, which provides inadequate coverage for healthcare costs, makes this difficult to accomplish.

Children treated with gene therapy represent another unique group with medical complexity. As gene therapy continues to revolutionize the treatment landscape for previously fatal pediatric conditions such as Spinal Muscular Atrophy and Duchenne Muscular Dystrophy, the financial burden on families has increased significantly (47, 48). The high costs associated with these cutting-edge, potentially curative therapies, place significant strain on pediatric patients and their families, often necessitating relocation of the families so that they may access specialized gene therapy centers. Addressing reimbursement inequities is crucial to ensure that all children, regardless of the complexity of their conditions, have equitable access to these life-changing treatments. Advancements in pediatric medicine led to the creation of patient populations with chronic and complex needs who require a higher level of coordinated care than ever before. It is imperative that key stakeholders commit to supporting the systems, providers, and families to help these children reach their full potential.

## Healthcare payment models need to address the full spectrum of pediatric care

The current payment models utilize a framework for adult healthcare that fundamentally does not work in its application to pediatrics. The U.S. Healthcare systems rely heavily on a Relative Value Unit (RVU) system to determine reimbursement for patient care. The RVU system was established by the Centers for Medicare and Medicaid Services (CMS) and plays a critical role in standardizing reimbursement across public and private payors. It assigns values to specific Current Procedural Terminology (CPT) codes, incorporating physician work, practice expenses, and malpractice considerations (49). However, this system, while useful for standardizing payment across various medical fields, falls short in accurately reflecting the complexities and scope of pediatric medical care. There are numerous aspects of pediatric care and procedures that are unique to children for which a CPT code does not even exist (50).

The inadequacy of the current healthcare model to address pediatric care is in part due to the lack of representation at the tables where these decisions are made. The current RVU system values are determined by the Relative Value Scale (RVS) Update Committee. This committee is comprised of representatives from various medical specialties, but only one seat is allocated to a pediatrician and is often filled by an adult subspecialist counterpart (51). The lack of representation of pediatrics, coupled with the fact that many existing pediatric-specific CPT codes have remained stagnant or untouched for decades, are major drivers of the systemic undervaluation of pediatric care (52). Furthermore, as a response to the escalating healthcare costs in the U.S., the RVU system has undergone revisions that disadvantage pediatric providers. An element of “net neutrality” has been incorporated into the system, meaning any increase in the RVU service value automatically triggers a decrease in the RVU valuation for another clinical service. This policy creates barriers to creating specific CPT codes or modifiers that address the unique needs of pediatric care.

The RVU model also fails to account for the variability in complexity and time spent on pediatric conditions, which range from common illnesses to rare congenital disorders. This diverse range of care requires varying amounts of time, resources, and education of caregivers, which is not accurately reflected in the RVU system. Additionally, many crucial aspects of pediatric care, such as developmental monitoring, behavioral health management, and chronic disease management, are non-procedural and undervalued in a system that predominantly favors procedural interventions. Preventive services, a primary component of pediatric care that includes vaccinations, developmental screenings, and health promotion activities, are also undervalued due to the RVU model’s focus on procedures. Moreover, the RVU model fails to appropriately value additional services integral to pediatric care that are not traditionally considered medical procedures. Examples of non-traditional medical care that are important to support health and development in children include school therapy, which supports children with special needs or chronic conditions in their educational settings, and child life services, which helps children and families cope with the stress and procedures during hospitalization. These services directly impact the health and well-being of children, but their value is not recognized in the RVU system (53). The limitations of the RVU model highlight the need for a more comprehensive approach to valuing pediatric services.

To address these issues, it is crucial to adjust billing, coding, and work relative value units to more accurately capture the complexities of pediatric care. Equally important is ensuring that pediatricians are included in decision-making groups and in setting standards. As pediatric medicine continues to evolve, it is essential that those familiar with the specific needs and complexities of pediatric care are engaged to ensure that the measurements for care are commensurate with the level of care required.

## Discussion

**Figure 1:**
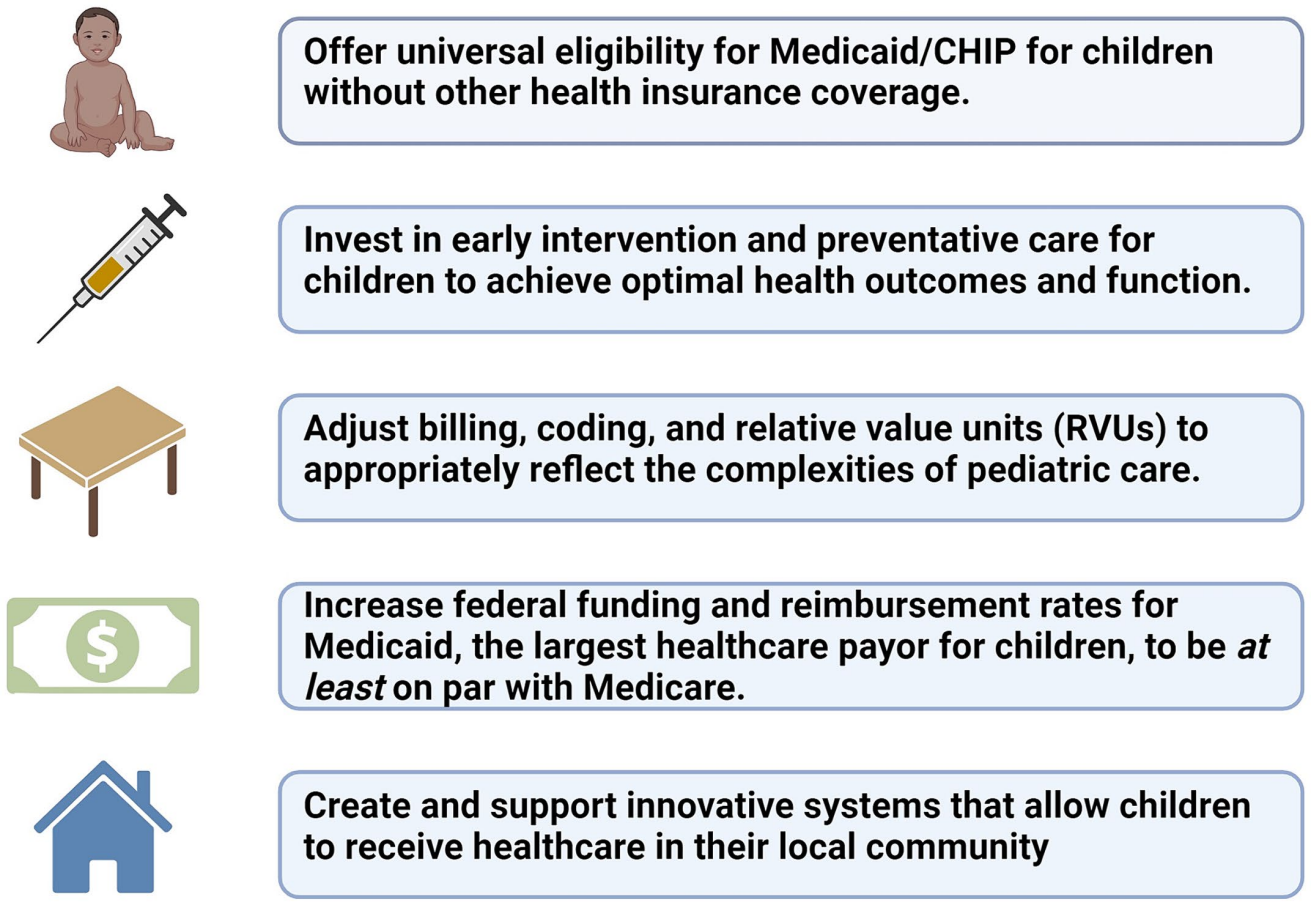
Call to action. Created with BioRender.com

Actions must be taken to improve the current state of pediatric care through modifications to financial policy and current healthcare systems. Although healthcare administrators and physicians arguably have an ethical obligation to provide services to patients, they must balance these uncompensated costs with financial pressures to maintain healthcare organizations. Issues such as delaying care to obtain prior authorization for medical services or providing nonemergent care to patients without insurance can cause moral distress to healthcare administrators (54). The healthcare system, particularly surrounding reimbursement, need to fundamentally change so that administrators and providers are incentivized to do what is best for the patient. The following changes would improve access to care for children, increase reimbursement for pediatric care in healthcare systems, ensure preventive and supportive care within home communities, and overall improve children’s health and wellbeing (Figure 1).

- **Offer universal eligibility for Medicaid/CHIP for children without other health insurance coverage.** Medicaid and CHIP coverage should start at birth and provide continuous eligibility periods to decrease lapses in coverage. Lack of insurance can result in large out-of-pocket costs for patients and families and gaps in healthcare, which can lead to underutilization of essential health services and worse outcomes.
- **Invest in early intervention and preventative care for children to achieve optimal health outcomes and function.** Increased funding is required both to support existing screening programs and invest in research to improve early detection and treatment for other fatal or severely debilitating diseases, including mental health-related disorders.
- **Adjust billing, coding, and relative value units (RVUs) to appropriately reflect the complexities of pediatric care.** Stakeholders and decision-making bodies should ensure that pediatricians are included in these decisions and setting the standards for RVU values. As pediatric medicine continues to evolve, pediatricians familiar with the complexities and uniqueness of pediatric care must engage in developing new pediatric-specific CPT codes and making sure the assigned values accurately reflect the level of care required.
- **Increase federal funding and reimbursement rates for Medicaid, the largest healthcare payor for children, to be *at least* on par with Medicare.** Medicaid reimbursement rates vary, with some states as low as 70% below Medicaid rates. Reimbursement directly affects the ability of hospitals, pediatricians, and subspecialists to care for children, which contributes to disparities in access to medical care. We echo the call by the American Academy of Pediatrics and National Academies Sciences, Engineering, and Medicine to increase Medicaid payment to incentivize healthcare institutions to prioritize care for children (55, 56).
- **Create and support innovative systems that allow children to receive healthcare in their local community.** Easily accessible medical homes need to be created to provide care based on national standards that promote high-quality preventative and acute care and help children avoid hospitalization. This care should be covered by Medicaid and CHIP.

The continuation of high-level healthcare is under threat for children in the United States. Children’s interests are not adequately represented in policy formation, given their lack of both the power of the purse and the ballot box. The long-standing undervaluing of children’s healthcare has resulted in decreased access to care for children across the U.S. and will have long-lasting negative impacts on society as children age into adulthood with increasingly preventable problems. We join other major medical organizations and encourage other key stakeholders to call for the prioritization of children’s health by increasing Medicaid coverage and payment to be on par with Medicare and ensure that pediatricians have a seat at the table where healthcare policies are made.

## Data availability statement

The original contributions presented in the study are included in the article/supplementary material, further inquiries can be directed to the corresponding author.

## Author contributions

JW: Writing – original draft, Writing – review & editing. AS: Writing – original draft. HG: Writing – original draft. SG: Writing – original draft. CP: Writing – original draft. AK: Writing – original draft. SH: Writing – original draft. JH: Writing – original draft. AB: Writing – original draft. JS: Writing – original draft. EL: Writing – original draft. KB: Conceptualization, Supervision, Writing – original draft, Writing – review & editing.
